# Alkaline stress and iron deficiency regulate iron uptake and riboflavin synthesis gene expression differently in root and leaf tissue: implications for iron deficiency chlorosis

**DOI:** 10.1093/jxb/erw328

**Published:** 2016-09-07

**Authors:** En-Jung Hsieh, Brian M. Waters

**Affiliations:** Department of Agronomy and Horticulture, University of Nebraska-Lincoln, Lincoln, NE 68583-0915, USA

**Keywords:** Bicarbonate, cucumber, *fefe* mutant, iron deficiency chlorosis, iron uptake, melon, shoot-to-root signaling.

## Abstract

Iron (Fe) is an essential mineral that has low solubility in alkaline soils, where its deficiency results in chlorosis. Whether low Fe supply and alkaline pH stress are equivalent is unclear, as they have not been treated as separate variables in molecular physiological studies. Additionally, molecular responses to these stresses have not been studied in leaf and root tissues simultaneously. We tested how plants with the Strategy I Fe uptake system respond to Fe deficiency at mildly acidic and alkaline pH by measuring root ferric chelate reductase (FCR) activity and expression of selected Fe uptake genes and riboflavin synthesis genes. Alkaline pH increased cucumber (*Cucumis sativus* L.) root FCR activity at full Fe supply, but alkaline stress abolished FCR response to low Fe supply. Alkaline pH or low Fe supply resulted in increased expression of Fe uptake genes, but riboflavin synthesis genes responded to Fe deficiency but not alkalinity. Iron deficiency increased expression of some common genes in roots and leaves, but alkaline stress blocked up-regulation of these genes in Fe-deficient leaves. In roots of the melon (*Cucumis melo* L.) *fefe* mutant, in which Fe uptake responses are blocked upstream of Fe uptake genes, alkaline stress or Fe deficiency up-regulation of certain Fe uptake and riboflavin synthesis genes was inhibited, indicating a central role for the FeFe protein. These results suggest a model implicating shoot-to-root signaling of Fe status to induce Fe uptake gene expression in roots.

## Highlight

Alkaline stress disrupts iron deficiency responses in *Cucumis* species, implicating interference of shoot-to-root iron status signaling.

## Introduction

Iron (Fe) is an important micronutrient that plays crucial roles in plant growth, development, and reproduction (Walker and Waters, 2011; Kobayashi and Nishizawa, 2012; Vigani *et al.*, 2013). Iron uptake into roots of graminaceous plant species (known as Strategy II) is characterized by production and secretion of phytosiderophores that chelate Fe(III) for uptake (Kobayashi and Nishizawa, 2012). Iron uptake by non-graminaceous angiosperm species (known as Strategy I) is characterized by rhizosphere acidification by H^+^-ATPase proteins, reduction of Fe(III) to Fe(II) by ferric chelate reductase (FCR) proteins, and uptake of Fe(II) by iron transporter proteins (Kobayashi and Nishizawa, 2012). The activity of these Fe uptake proteins is up-regulated in Fe-deficient roots.

Many molecular components of the Strategy I Fe uptake system have been identified. *FERRIC REDUCTION OXIDASE 2* (*FRO2*) encodes the primary root surface FCR in Arabidopsis (Robinson *et al.*, 1999), and the corresponding genes in cucumber (*Cucumis sativus* L.) and melon (*Cucumis melo* L.) are called *FRO1* (Waters *et al.*, 2007, 2014). Iron (II) transporter genes include *IRT1* (Eide *et al.*, 1996; Varotto *et al.*, 2002; Mukherjee *et al.*, 2006) and *NRAMP1* (Curie *et al.*, 2000; Thomine *et al.*, 2000; Cailliatte *et al.*, 2010). *AtFRO2, AtIRT1*, *AtNRAMP1*, and numerous other genes are up-regulated under Fe deficiency by the basic helix–loop–helix (bHLH) transcription factor AtFIT (Colangelo and Guerinot, 2004; Jakoby *et al.*, 2004; Yuan *et al.*, 2005), a homolog of the tomato FER protein (Ling *et al.*, 2002; Colangelo and Guerinot, 2004). *AtFIT* gene expression is typically up-regulated by Fe deficiency (Colangelo and Guerinot, 2004; Jakoby *et al.*, 2004; Lucena *et al.*, 2006). A group of four closely related Arabidopsis bHLH genes, *AtbHLH38*, *AtbHLH39*, *AtbHLH100*, and *AtbHLH101*, are classified in clade Ib of the *bHLH* superfamily (Wang *et al.*, 2007). The AtFIT protein regulates expression of its target genes as a heterodimer complex of AtFIT and a clade Ib bHLH protein (Colangelo and Guerinot, 2004; Yuan *et al.*, 2008; Wang *et al.*, 2013). The melon *C940-fe* mutant (*fefe*) (Nugent and Bhella, 1988, Nugent, 1994) is similar to the *fit* mutant, in that it does not up-regulate FCR activity and rhizosphere acidification under Fe deficiency, and is chlorotic (Nugent, 1994). Our characterization of the *fefe* mutant showed that 82 genes that were up-regulated by Fe deficiency in wild-type roots were not regulated by Fe deficiency in the *fefe* mutant, including key Fe uptake genes (Waters *et al.*, 2014). Thus, both *fit* and *fefe* mutants have blocked Fe uptake responses upstream of Fe uptake genes.

Less is known about molecular Fe deficiency responses in leaves than in roots, as only a few studies have profiled genome-wide gene expression in leaves (Waters *et al.*, 2012; Ivanov *et al.*, 2012; Rodríguez-Celma *et al.*, 2013*a*; Moran Lauter *et al.*, 2014). Several Fe regulated genes respond to Fe deficiency in both leaf and root tissues, whereas some are specific to roots or leaves. While *AtFIT* is only expressed and regulated by Fe in roots, the transcripts of *AtbHLH38*, *AtbHLH39*, *AtbHLH100*, and *AtbHLH101* are up-regulated in both roots and leaves of Fe-deficient Arabidopsis (Wang *et al.*, 2007; Rodríguez-Celma *et al.*, 2013*a*). In our previous Arabidopsis leaf microarray study (Waters *et al.*, 2012), At1G47400 (named iron responsive protein 1 (*AtIRP1*) in Rodríguez-Celma *et al.*, 2013*a*) and *AtKCS17* (3-ketoacyl-CoA synthase, At4G34510) were among the most strongly up-regulated genes in Fe-deficient Arabidopsis leaves (10.2-fold for *AtIRP1* and 36.0-fold for *AtKCS*, respectively). A more detailed knowledge of leaf Fe deficiency responses is needed to understand whole-plant adaptations to low Fe conditions, since a leaf-originated signal is thought to be necessary for normal regulation of Fe deficiency responses in roots (De Nisi *et al.*, 2012; García *et al.*, 2013).

Although Fe is abundant in soils, it has low solubility, especially in calcareous, alkaline soils, which occur on 30% of the earth (Chen and Barak, 1982). Plants can show iron deficiency chlorosis (IDC) on alkaline soils (Mengel and Geurtzen, 1986), resulting in reduced growth and yield (Hansen *et al.*, 2004; Rogovska *et al.*, 2007; Briat *et al.*, 2015). Uptake of other metal micronutrients, such as Mn and Zn, can also be inhibited in alkaline soils (George *et al.*, 2012). Soil alkalinity is largely due to bicarbonates (HCO_3_^−^) and carbonates (CO_3_^2−^) (Coulombe *et al.*, 1984; Mengel *et al.*, 1984), and therefore, bicarbonate has been commonly used to induce IDC symptoms in hydroponic Fe nutrition studies (Coulombe *et al.*, 1984; Chaney *et al.*, 1992; Romera *et al.*, 1992*a*; Lin *et al.*, 1997; Waters and Troupe, 2012). However, whether IDC results from low Fe supply, alkaline stress, or a combination of these factors is still unclear. Most IDC studies to date have not treated Fe supply and alkalinity as separate variables. Studies that applied bicarbonate to both Fe-deficient and Fe-sufficient plants (Fleming *et al.*, 1984; Romera *et al.*, 1992*a*; Alcántara *et al.*, 2000) were carried out prior to modern molecular methods, and thus it is not clear whether low Fe supply and alkaline stress cause equivalent molecular responses. In Strategy I species, bicarbonate-treated Fe-deficient plants had low root expression of *FIT*, *FRO2*, and *IRT1*, and had inhibited root FCR activity compared with Fe-deficient plants grown without bicarbonate (Romera *et al.*, 1997; Lucena *et al.*, 2007; García *et al.*, 2014). However, we found that cucumber FCR activity was stimulated by bicarbonate treatment in plants supplied with Fe (Waters and Troupe, 2012). Except for Romera *et al.* (1992*a*), pH-matched control treatments have not been included in these studies. In pilot studies in our lab with sodium bicarbonate, potassium bicarbonate, and HEPES buffer, the FCR activity response of plants in pH buffered treatments was not distinguishable from the FCR response of bicarbonate-treated plants, regardless of the counter-ion. As such, we use the term alkalinity to refer to bicarbonate treatments. One of our objectives was to determine how pH influences physiological and molecular responses to Fe deficiency in roots and leaves.

Iron deficient Strategy I plant species have long been known to increase efflux of root exudates (Cesco *et al.*, 2010). Some species, such as *Arabidopsis thaliana*, produce phenolic compounds (Fourcroy *et al.*, 2014; Schmid *et al.*, 2014; Schmidt *et al.*, 2014) while other species, including cucumber and melon, produce flavin compounds (Susin *et al.*, 1993; Welkie, 2000; Rodríguez-Celma *et al.*, 2011*b*). Although the function of flavin compounds in plant Fe deficiency is not well defined, they may function in reduction or complexation of extracellular Fe to facilitate Fe acquisition (Cesco *et al.*, 2010; Sisó‐Terraza *et al.*, 2016). Proteins involved in riboflavin synthesis increased in abundance in response to Fe deficiency or Fe deficiency in alkaline conditions (Rellán-Álvarez *et al.*, 2010; Rodríguez-Celma *et al.*, 2011*b*) and genes involved in riboflavin biosynthesis were up-regulated in iron-deficient roots in normal or alkaline conditions (Rellán-Álvarez *et al.*, 2010; Rodríguez-Celma *et al.*, 2013*b*). However, the expression of riboflavin synthesis genes by alkaline stress separately from Fe deficiency has not been studied. Thus, another objective was to determine how riboflavin synthesis genes respond to Fe deficiency and alkaline stress in roots and leaves.

We addressed the objectives above using molecular and physiological approaches, by measuring leaf chlorosis, root FCR activity, Fe and other metal micronutrient accumulation, and expression of Fe uptake genes and riboflavin synthesis genes in cucumber roots and leaves. Our third objective was to use the melon *fefe* mutant to determine if alkaline-stimulated root gene expression depends on the *fefe* Fe deficiency regulatory pathway. The results of this research will lead to increased understanding of how alkaline stress inhibits Fe uptake and causes IDC, and will allow improved design of future studies to develop alkaline stress-tolerant crop varieties.

## Materials and methods

### Plant materials and growth conditions

Cucumber seeds of cv Ashley were purchased from Eden Brothers (Asheville, NC, USA) and seeds of Miniature White were purchased from Jonny’s Selected Seeds (Winslow, ME, USA). Melon seeds of cv Edisto were purchased from Victory Seed Company (Molalla, OR, USA), and seeds of C940-fe (*fefe*; Nugent, 1994) were a gift from Michael A. Grusak (USDA-ARS Children’s Nutrition Research Center, Houston, TX, USA). Seeds were germinated in germination paper soaked with 0.1mM CaSO_4_ and incubated in the dark at 30 °C for 3 days. Seedlings were transferred to black tubs (four seedlings per tub) with 750mL of nutrient solution made with 1.5mM KNO_3_, 0.8mM Ca(NO_3_)_2_, 0.3mM (NH_4_)H_2_PO_4_, 0.2mM MgSO_4_, 25 μM CaCl_2_, 25 μM H_3_BO_3_, 2 μM MnCl_2_, 2 μM ZnSO_4_, 0.5 μM Na_2_MoO_4_, 0.1 μM CuSO_4_, and 1mM MES buffer (pH 5.5). Iron was supplied as Fe(III)–ethylenediamine-*N*,*N*′-bis(2-hydroxyphenylacetic acid) (EDDHA) (Sprint 138, Becker-Underwood) at concentrations indicated below. The Fe(III)–EDDHA chelate is stable at the mildly acidic and alkaline pH used in this study (Chaney *et al.*, 1972, Halvorson and Lindsay, 1972). Plants were grown in a growth chamber at 22 °C with a 16h photoperiod and photosynthetic photon flux density of 300 μmol m^−2^ s^−1^ photosynthetically active radiation. Bicarbonate was supplied as potassium bicarbonate (Fisher Scientific) or sodium bicarbonate (Arm & Hammer) with no difference in results. Plants were grown for 4 days in complete solution with 0.5, 1.0, 2.5, or 10 μM Fe (pretreatment), followed by 3 days of treatment with bicarbonate (10mM) at the same Fe concentration as during the pretreatment period. At the end of the treatment, the first true leaf was still growing and the second leaf was emerging (see Supplementary Fig. S1 at *JXB* online for plant DW). Final nutrient solution pH was determined using a pH meter at the end of the treatment period.

### Ferric chelate reductase activity and chlorophyll content

Ferric chelate reductase activity was measured using whole roots of individual cucumber plants after 3 days of treatment. Roots were excised, rinsed in deionized water, and submerged in 20ml assay solution (1mM MES buffer, pH 5.5, 150 μM Fe(III)–EDTA, and 200 μM ferrozine) for 30–60min. Ferrozine–Fe(II) was measured by absorbance at 562nm (subtracting blanks of assay solution with no plants) and reduced Fe was calculated using the extinction coefficient of 28.16mM cm^−1^. Chlorophyll of the first true leaf was determined using a SPAD-502 chlorophyll meter (Minolta). Significance of differences between Fe treatments at each bicarbonate level, and between bicarbonate treatments at each Fe level were determined by Student’s *t*-test.

### Quantification of dry weight and mineral content

Note that with Fe(III)EDDHA as an Fe source, Fe does not accumulate in the apoplast, in contrast to Fe sources such as Fe(III)EDTA (Longnecker and Welch, 1990; Strasser *et al.*, 1999) or FeSO_4_ (Waters and Blevins, 2000). Plants were dissected into roots, first true leaf, and the remainder of the plant (stem+cotyledons) after 3d treatments and dried at 70 °C in a drying oven. After measuring dry weight (DW), tissue samples were digested in concentrated nitric acid–hydrogen peroxide with stepwise heating at 100, 125, 150, and 165 °C to dryness, and then resuspended in 5ml 1% HNO_3_ (Guttieri *et al.*, 2015). Iron, Cu, Zn, and Mn contents for plant parts were quantified by inductively coupled plasma mass spectrometry in the University of Nebraska Redox Biology Center Spectroscopy Facility. Total plant DW or mineral content for each replicate plant was determined by summing plant parts. Significant differences between treatments for DW and each mineral were determined by one-way ANOVA.

### RNA preparation, RT-PCR and real-time PCR analyses

Total RNA was extracted from approximately 80mg of frozen tissues of cucumber or melon plants using the RNeasy Plant Mini Kit (Qiagen). RNA samples were treated with DNaseI (Promega, USA) and RNA quality and concentration were determined by *A*_260_/*A*_280_ ratio. For reverse transcription reactions, 3 μg of total RNA was used with the High Capacity cDNA Reverse Transcription Kit (Applied Biosystems, CA, USA). Single-stranded cDNA corresponding to 15ng of total RNA was used as a template in 15 μl total volume for real-time PCR assay. Real-time PCRs were carried out with 667nM of gene-specific primers and GoTaq qPCR Master Mix (Promega, USA) using an IQ5 MyiQ detection system (Bio-Rad, Hercules, CA, USA). Coding sequences for cucumber and melon *bHLH38*, *bHLH101*, *NRAMP1*, *RIBA1*, *PYRD*, *PHS1*, *DMRLs* and *F6’H1* (Table 1) were identified from Cucurbit Genomics Database (http://www.icugi.org/cgi-bin/ICuGI/index.cgi) or from the Melonomics database (https://melonomics.net/) by BLAST searches and phylogenetic analysis. Primer sequences (Supplementary Table S1) were designed using the NCBI primer design tool (http://www.ncbi.nlm.nih.gov/tools/primer-blast/index.cgi). The thermal cycler program was one initial cycle of 95 °C, 8min 30s; followed by cycles of 95 °C, 10s; 57 °C, 30s; 72 °C, 15s, with 40 cycles; followed by melt curve analysis for all genes. The relative gene expression considered the 10 μM Fe–0 bicarbonate-treated roots as control, and was calculated using the equation *Y*= $2^{-\Delta \Delta C_{\text{t}}},$ where

**Table 1.** *Riboflavin synthesis pathway gene IDs and descriptions in roots of cucumber* (Cucumis sativa), *melon* (Cucumis melo), *and Arabidopsis thaliana. Normalized read counts for wild-type melon Edisto and the fefe mutant roots at normal Fe supply (10 μM) and under no added Fe conditions are from* Waters et al. (2014). *Significant fold-changes (FC) in read counts in the Fe-deficient samples are shown in bold.*

Gene name	Cucumber ID	Melon ID	Arabidopsis ID	Description	WT +Fe	WT –Fe	WT FC	*fefe* +Fe	*fefe* –Fe	*fefe* FC
*RIBA1*	Csa4M111580	Melo3C024826	AT5G64300	GTP cyclohydrolase II	656	11 118	**17.0**	118	110	0.9
*PYRD*	Csa6M003430	MU51870	AT4G20960	Diaminohydroxyphosphoribosylamino- pyrimidine deaminase	369	505	1.4	415	394	0.9
*PHS1*	Csa1M655920	Melo3C010048	AT3G47390	Pyrimidine reductase	177	1670	**9.4**	27	25	1.0
*DMRLs*	Csa6M366300	MU59012	AT2G44050	Dimethyl-8-ribityllumazine synthase	1838	6234	**3.4**	1690	1142	0.7
*RIBC*	Csa6M128550	MU45607	AT2G20690	Riboflavin synthase α chain	220	385	1.8	302	233	0.8

$$\Delta \Delta C_{\text{t}}=\text{test gene}\;(C_{\text{t},\text{treatment}}-C_{\text{t},\text{control}})\;-\;\text{ubiquitn}(C_{\text{t},\text{treatment}}-C_{\text{t},\text{control}}).$$

At least three independent RNA extractions (from three different plants) and RT-qPCR reactions with two technical replicates per sample were performed. Significance of differences between Fe treatments at each bicarbonate level, and between bicarbonate treatments at each Fe level were determined by Student’s *t*-test.

## Results

### Physiological Fe deficiency and alkaline stress responses in cucumber

**Fig. 1.**
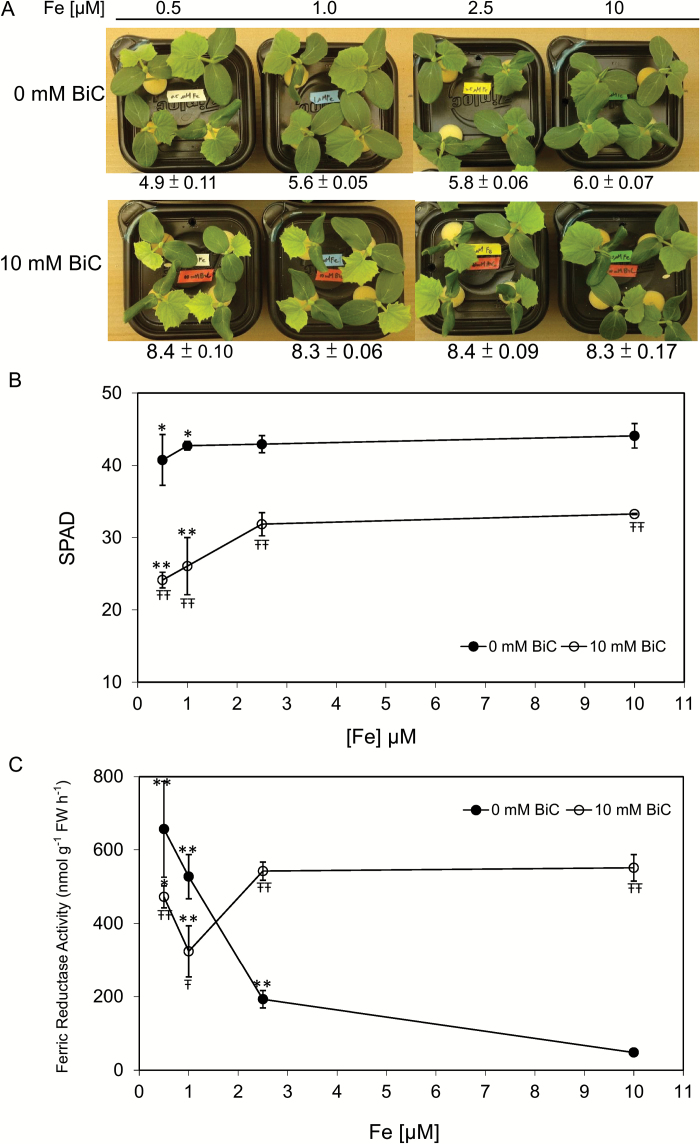
Cucumber (cv. Ashley) plant responses to Fe supply in normal pH or alkaline nutrient solution. Cucumber seedlings were pre-treated in hydroponic solution with 0.5, 1.0, 2.5, or 10 μM Fe for 3 days, and then supplied without or with 10mM bicarbonate (BIC) for 4 days. (A) Photograph of the plants in each treatment, with final solution pH (means±SD, *n*=6) indicated below photograph. (B) Chlorophyll level of first leaf as measured by Minolta SPAD chlorophyll meter. (C) Ferric chelate reductase activities of roots. * and ** indicate statistical significance of *P*<0.05 and *P*<0.01, respectively, compared with 10 μM Fe within each curve; Ŧ and ŦŦ indicate statistical significance of *P*<0.05, *P*<0.01, respectively, comparing 10mM bicarbonate with 0mM bicarbonate at each Fe supply.

To understand the plant response to Fe supply at normal hydroponic pH and at alkaline pH, we grew plants with or without bicarbonate over a range of Fe supply, using pH-stable Fe(III)–EDDHA to ensure that Fe was soluble at all pH treatments (Chaney *et al.*, 1972; Halvorson and Lindsay, 1972). Without bicarbonate, final solution pH was lower (pH 4.9) at the lowest Fe supply (0.5 μM) compared with final solution pH of the 10 μM Fe control (pH 6.0; Fig. 1A), likely reflecting increased H^+^-ATPase activity at low Fe supply. With bicarbonate, the final solution pH did not vary by Fe supply, and was 8.3–8.4 in all treatments. The first leaf of cucumber plants was slightly more chlorotic at the lowest Fe supply without bicarbonate, indicating that none of the Fe treatments caused severe Fe deficiency at normal pH. With alkaline stress (10mM bicarbonate), the chlorophyll content of the first leaf was significantly lower at each Fe concentration than in plants without bicarbonate (Fig. 1B). The root FCR activity was highest at 0.5 μM Fe supply without bicarbonate, and was also significantly up-regulated at 1.0 and 2.5 μM Fe (Fig. 1C). However, plants supplied with bicarbonate had similar FCR activity at all Fe concentrations. The FCR activity of plants treated with bicarbonate was significantly higher than that of plants without bicarbonate at 2.5 and 10 μM Fe, but was lower than that of plants without bicarbonate at 0.5 μM Fe. Thus, while low Fe supply and alkaline stress both resulted in elevated FCR activity in cucumber roots, the patterns were quite different. Comparing bicarbonate treatments at specific Fe supplies, bicarbonate caused either inhibition (0.5 μM Fe) or stimulation (10 μM Fe) of FCR activity. We obtained similar chlorophyll and FCR activity results with another variety of cucumber (Miniature White; Supplementary Fig. S2). These results indicated that alkaline stress elevated the root FCR activity relative to Fe-replete plants, but abolished the normal response to Fe supply.

**Fig. 2.**
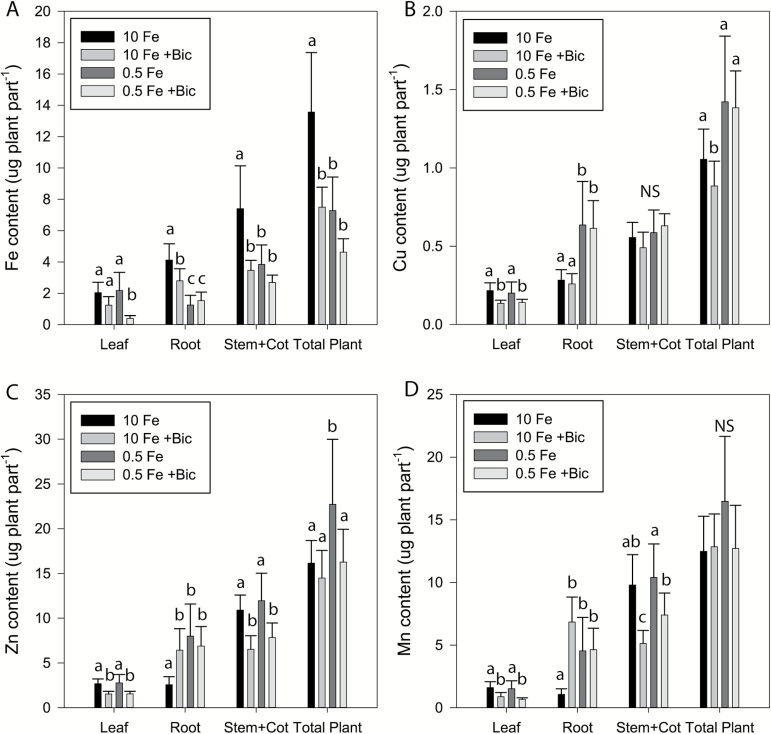
Mineral content in cucumber (cv. Ashley) plants. Cucumber seedlings were transferred into hydroponics with 0.5 or 10 μM Fe for 4 days pretreatment, and then treated with or without 10mM bicarbonate for 3 days. The roots, first leaf, and stem+cotyledons of each plant were harvested separately. (A) Fe content, (B) Cu content, (C) Zn content, and (D) Mn content in different parts of plants and whole plants. Bars represent mean±SD (*n*=8). Different letters indicate significant differences (*P*<0.05) based on ANOVA using the Holm–Sidak method; NS indicates no significant differences between treatments.

For an additional comparison of plant response to Fe deficiency and alkaline stress, we measured plant biomass and mineral accumulation in plant tissues. Biomass of the total plant or plant parts was not significantly affected by the treatments, except for roots of 0.5 Fe–10mM bicarbonate-treated plants, which were slightly larger than roots of the other treatments (Supplementary Fig. S1). The control plants grown with 10 μM Fe had the highest total Fe content, which decreased by about half under mild Fe deficiency (0.5 μM Fe; Fig. 2A). Bicarbonate treatment at 10 μM Fe supply resulted in an approximately 40% decrease in total Fe content, indicating that alkaline stress inhibited Fe uptake, despite using a pH-stable Fe source. The low Fe, bicarbonate-treated plants had the lowest Fe content in leaves, at 19% of the control value. Iron content decreased in roots and stem+cotyledon with low Fe and/or bicarbonate treatment (Fig. 2A). For other metal micronutrients, Cu, Zn, and Mn contents decreased in leaf tissue of bicarbonate-treated plants in both Fe supply regimes (Fig. 2B–D). In stem+cotyledon, Zn and Mn decreased under bicarbonate treatment, while Cu content was unchanged. In root, Mn and Zn increased in low Fe and bicarbonate treatments, while Cu increased only in low Fe treatments.

### Molecular responses to Fe deficiency and alkaline stress in cucumber

**Fig. 3.**
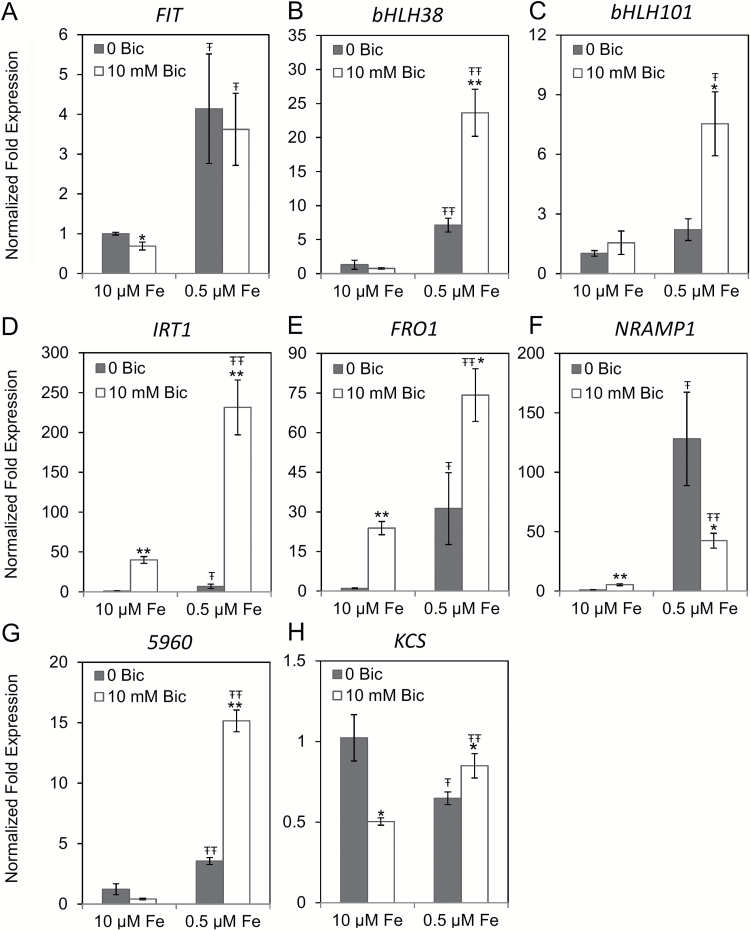
Quantitative RT-PCR analyses of transcript levels of Fe uptake-related or Fe regulated genes in cucumber (cv. Ashley) roots after 3 days of treatment. The results were normalized to ubiquitin as an internal reference. The transcript abundance of each gene (means±SE, *n*=3) was normalized to roots of the control treatment (10 μM Fe and 0mM bicarbonate). * and ** indicate statistical significance at *P*<0.05 and *P*<0.01, respectively, comparing bicarbonate treatments within the same Fe supply; Ŧ and ŦŦ indicate statistical significance at *P*<0.05 and *P*<0.01, respectively, comparing different Fe supply within the same bicarbonate treatment.

To gain a molecular understanding of similarities and differences between molecular responses to Fe deficiency and alkaline stress, we measured gene expression of known Fe deficiency responsive genes in root and first leaf tissue of cucumber plants. The Fe uptake transcription factor genes *CsFIT* and *CsbHLH38* were up-regulated by Fe deficiency in roots (Fig. 3A, B), but *CsFIT* was not further induced by bicarbonate treatment at low Fe and was not induced by bicarbonate at replete Fe. Transcripts of *CsbHLH38* and *CsbHLH101* were greatly induced by bicarbonate in roots with low Fe supply, but there was no stimulation by bicarbonate in roots with normal Fe supply (Fig. 3B, C). The expression of *CsIRT1* and *CsFRO1* was increased under Fe deficiency (Fig. 3D, E), and their expression was increased further by bicarbonate at low Fe concentration. Bicarbonate also stimulated expression of these genes at normal Fe supply. In contrast, while the expression of *CsNRAMP1* was strongly induced by Fe deficiency (Fig. 3F), bicarbonate treatment at low Fe supply resulted in much lower induction compared with the treatment without bicarbonate. Two additional genes based on previous leaf microarray results from Fe-deficient Arabidopsis (Waters *et al.*, 2012) were studied. The *5960* gene (Csa2M005960, homologous to *AtIRP1*) had an expression pattern similar to *bHLH38*. The *CsKCS* gene (Csa2M361630) was not consistently regulated by Fe or bicarbonate in roots, although some treatments were statistically significant.

**Fig. 4.**
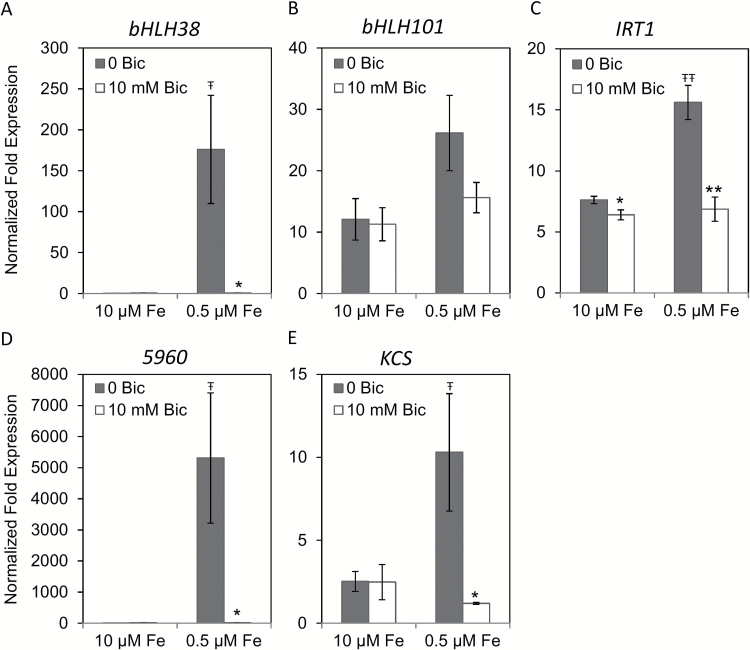
Quantitative RT-PCR analyses of transcript levels of Fe uptake-related or Fe regulated genes in cucumber (cv. Ashley) first leaf tissue after 3 days of treatment. The results were normalized to ubiquitin as an internal reference. The transcript abundance of each gene (means±SE, *n*=3) was normalized to transcript abundance in roots of the control treatment (10 μM Fe and 0mM bicarbonate). * and ** indicate statistical significance at *P*<0.05 and *P*<0.01, respectively, comparing bicarbonate treatments within the same Fe supply; Ŧ and ŦŦ indicate statistical significance at *P*<0.05 and *P*<0.01, respectively, comparing different Fe supply within the same bicarbonate treatment.

In leaf tissue, two patterns of gene expression were apparent, both of which were substantially different from root gene expression patterns. *CsFIT*, *CsFRO2*, or *CsNRAMP1* transcripts were not detected in leaf tissue. None of the other genes’ expression increased in leaf in response to bicarbonate at full Fe supply (Fig. 4). The transcripts of *CsbHLH38* and *Cs5960* were highly induced in leaf tissue under Fe deficiency, but this induction was completely abolished in the leaf of bicarbonate-treated plants (Fig. 4A, D). However, these transcripts were synergistically increased by Fe deficiency and alkaline stress in roots (Fig. 3B, G). *CsbHLH101* and *CsIRT1* were induced by Fe deficiency, to a lower extent than in roots, but the addition of bicarbonate diminished their expression in the Fe-deficient leaf to control levels (Fig. 4B, C), whereas their expression was stimulated in roots (Fig. 3C, D). The expression of *CsKCS* was induced by Fe deficiency in leaf, but not in root (Figs. 3H and 4E). Thus, alkaline stress interfered with the molecular leaf response to low Fe supply.

### Expression of riboflavin synthesis pathway genes

**Fig. 5.**
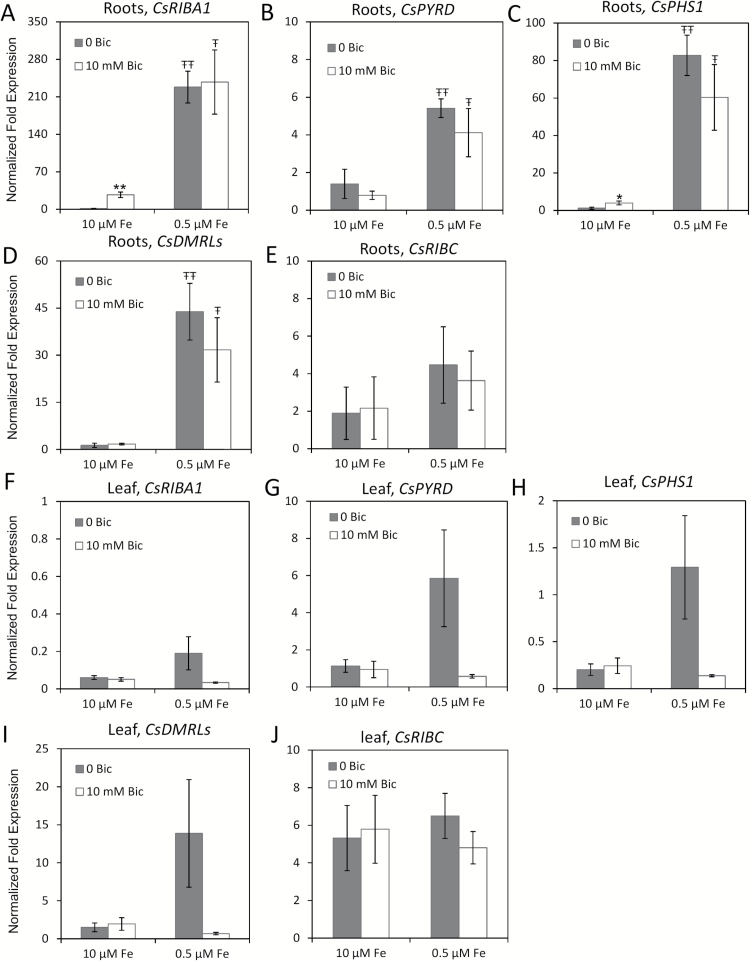
Quantitative RT-PCR analyses of riboflavin synthesis genes in cucumber (cv. Ashley) roots (A–E) and first leaf (F–J) after 3 days of treatment. The results were normalized to ubiquitin as an internal reference. The transcript abundance of each gene (means±SE, *n*=3) was normalized to transcript abundance in roots of the control treatment (10 μM Fe and 0mM bicarbonate). * and ** indicate statistical significance at *P*<0.05 and *P*<0.01, respectively, comparing bicarbonate treatments within the same Fe supply; Ŧ and ŦŦ indicate statistical significance at *P*<0.05 and *P*<0.01, respectively, comparing different Fe supply within the same bicarbonate treatment.

We identified homologs of genes known to be involved in the riboflavin synthesis pathway (Table 1) and measured their gene expression in response to Fe deficiency and alkaline stress. The riboflavin synthesis genes were up-regulated by Fe deficiency in roots, except for *CsRIBC* (Fig. 5). The addition of bicarbonate to the low Fe treatment did not greatly change the transcript levels, but *CsRIBA1* and *CsPHS1* riboflavin synthesis genes were significantly induced by bicarbonate under 10 μM Fe supply. Thus, plant Fe status seems to be a more important factor than pH for induction of riboflavin synthesis genes, in contrast to the Fe uptake genes. In the leaf, none of the riboflavin synthesis genes were up-regulated by bicarbonate treatment at full Fe supply. Three of the genes were up-regulated by Fe deficiency, but this up-regulation was abolished when bicarbonate treatment was also applied (Fig. 5F–J), similar to what we observed for other leaf genes (Fig. 4).

### Alkaline stress responses in *fefe* melon plants

**Fig. 6.**
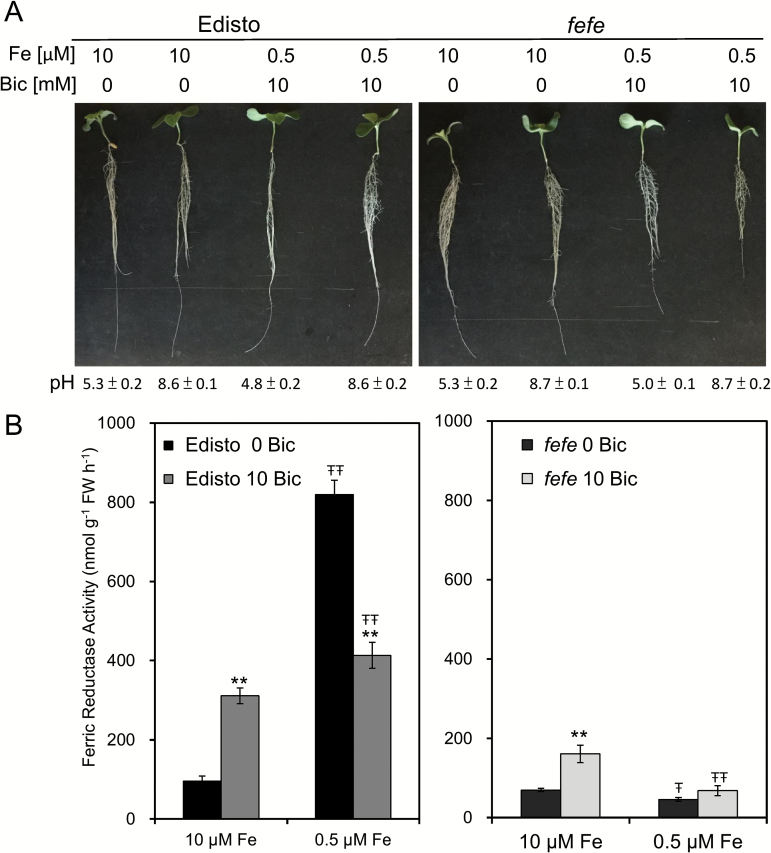
Melon plant growth and ferric chelate reductase activity with Fe deficiency and bicarbonate treatments. Wild-type (Edisto) and *fefe* mutant seedlings were grown in hydroponics with 0.5 or 10 μM Fe for pretreatment for 4 days, and then supplied without or with 10mM bicarbonate for 3 days. (A) Photograph of the plants. The final nutrient solution pH of each treatment (means±SE, *n*=6) is indicated below each plant. (B) Ferric chelate reductase activities of roots after 4 days. * and ** indicate statistical significance at *P*<0.05 and *P*<0.01, respectively, comparing bicarbonate treatments within the same Fe supply; Ŧ and ŦŦ indicate statistical significance at *P*<0.05 and *P*<0.01, respectively, comparing Fe supply treatments within the same bicarbonate treatment.

Since our results with cucumber indicated that plant Fe uptake genes were up-regulated by both alkaline stress and Fe deficiency, we tested whether up-regulation of their melon counterparts requires a functional *FeFe* gene. The WT line Edisto was not strongly chlorotic under low Fe (0.5 μM Fe) or alkaline pH (10mM bicarbonate) stress. The *fefe* mutant was more sensitive to low Fe supply, and *fefe* roots had a yellow coloration under low Fe with bicarbonate (Fig. 6A, right). Edisto FCR activity was stimulated by bicarbonate at 10 μM Fe supply. FCR activity was stimulated by Fe deficiency (0.5 μM), but this activity was repressed by adding bicarbonate to the low Fe treatment (Fig. 6B, left). FCR activity was not up-regulated in *fefe* melon at low Fe supply, but FCR was significantly stimulated by bicarbonate at 10 μM Fe, but to a lower extent than in Edisto (Fig. 6B, right). This indicated that alkaline stress stimulation of FCR activity is not entirely dependent on the FeFe protein.

**Fig. 7.**
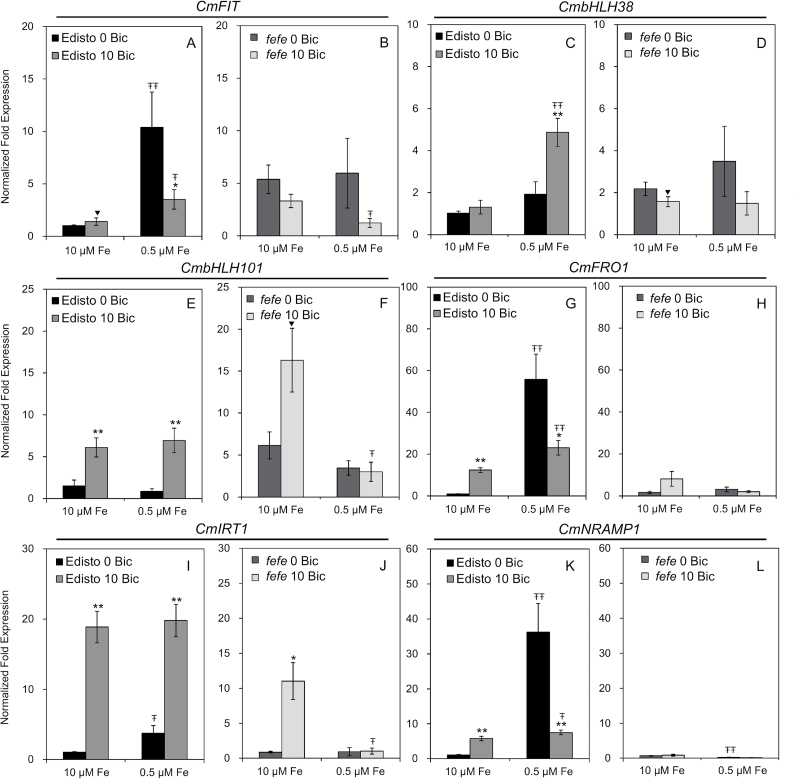
Quantitative RT-PCR analyses of Fe uptake related genes in roots of wild-type Edisto (A, C, E, G, I, and K) and *fefe* mutant (B, D, F, H, J, and L) after 3 days of treatment. The results were normalized to ubiquitin as an internal reference. The transcript abundance of each gene (means±SE, *n*=3) of both genotypes was normalized to Edisto root samples of the control treatment (10 μM Fe and 0mM bicarbonate). * and ** indicate statistical significance at *P*<0.05 and *P*<0.01, respectively, comparing bicarbonate treatments within the same Fe supply; Ŧ and ŦŦ indicate statistical significance at *P*<0.05 and *P*<0.01, respectively, comparing different Fe supply within the same bicarbonate treatment.

In the WT roots, the *CmFIT* gene was up-regulated by Fe deficiency, but its expression was greatly reduced by bicarbonate treatment (Fig. 7A). *CmbHLH38* was induced by bicarbonate in WT roots at low Fe supply (Fig. 7C), and *CmbHLH101* was induced by bicarbonate at both low Fe and high Fe supply (Fig. 7E). The expression pattern of *CmFRO1* corresponded to FCR activity in WT melon (Figs. 6B and 7G). The transcript patterns of both *CmIRT1* and *CmNRAMP1* in Edisto were consistent with expression in cucumber (Figs 2D, F and 7I, K). In *fefe* roots, *CmFIT*, *CmbHLH38*, *CmFRO1*, and *CmNRAMP1* did not respond to Fe deficiency or to bicarbonate treatment. However, both *CmbHLH101* and *CmIRT1* had higher expression in bicarbonate-treated, Fe-sufficient roots. These results suggested that the alkaline stress response was mostly, but not entirely, dependent on *fefe*.

### Expression of riboflavin synthesis pathway genes in *fefe* melon

**Fig. 8.**
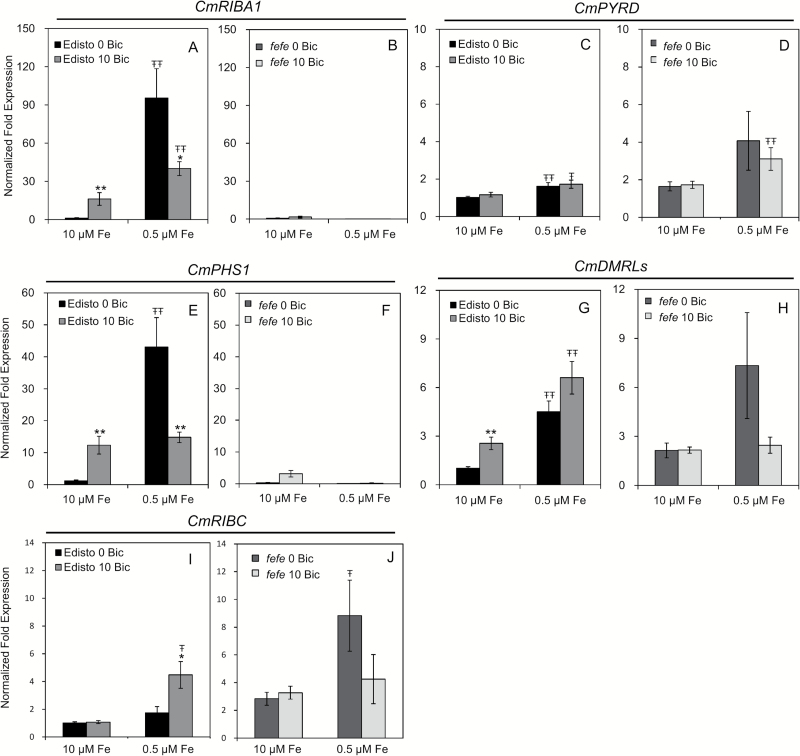
Quantitative RT-PCR analyses of riboflavin synthesis genes in roots of wild-type Edisto (A, C, E, G, and I) and *fefe* mutant (B, D, F, H, and J) after 3 days of treatment. The results were normalized to ubiquitin as an internal reference. The transcript abundance of each gene (means±SE, *n*=3) of both genotypes was normalized to Edisto root samples of the control treatment (10 μM Fe and 0mM bicarbonate). * and ** indicate statistical significance at *P*<0.05 and *P*<0.01, respectively, comparing bicarbonate treatments within the same Fe supply; Ŧ and ŦŦ indicate statistical significance at *P*<0.05 and *P*<0.01, respectively, comparing different Fe supply within the same bicarbonate treatment.

To test whether Fe deficiency regulation of riboflavin synthesis genes requires the *FeFe* gene, we gathered RNAseq expression data from our previous study (Waters *et al.*, 2014) (Table 1). In the WT, three (*RIBA1*, *PHS1*, and *DMRLs*) of the five genes were significantly up-regulated under Fe deficiency, but none of these genes were up-regulated by Fe deficiency in the *fefe* mutant. To test whether up-regulation of these genes in response to alkaline stress depends on the *FeFe* gene, we measured their expression in WT and *fefe* mutant roots by RT-qPCR. Consistent with the RNAseq results, *CmRIBA1*, *CmPHS1*, and *CmDMRLs* were induced by Fe deficiency in WT. Transcript levels of *CmRIBA1* and *CmPHS1* were lower in bicarbonate-treated Fe-deficient Edisto roots (Fig. 8A, C), similar to *CmFIT* expression, but *CmDMRLs* expression was increased (Fig. 8G). In the *fefe* mutant, expression of *CmPYRD* was somewhat higher in low Fe, both with or without bicarbonate treatment, and *CmDMRLs* was up-regulated under low Fe without bicarbonate (Fig. 8). However, expression of *CmRIBA1* and *CmPHS1* was almost completely abolished in the *fefe* mutant, suggesting that expression of these genes strongly depends on the *FeFe* regulatory pathway.

## Discussion

Although it is well known that IDC occurs in alkaline soils with low Fe availability, the physiological and molecular basis for this phenomenon is not well understood. In this study, we treated Fe supply and pH as separate variables. Using a range of Fe supply with two nutrient solution pHs, we found that root FCR activity responded to Fe supply only at normal pH, not at alkaline pH (Fig. 1C). We then used low and normal Fe supply, with or without bicarbonate, to determine whether gene expression in roots and leaves responded to Fe supply at each pH, and whether gene expression responded to pH at each Fe supply. Whether root FCR activity is inhibited or induced by alkaline pH depends on the point of reference. At low Fe supply, bicarbonate treatment inhibited FCR activity, relative to the treatment without bicarbonate. However, at alkaline pH the FCR activity at 10 and 2.5 μM Fe was elevated, relative to activity at normal pH. We found similar results for melon FCR activity (Fig. 6B), in that bicarbonate treatment inhibited FCR activity in Fe-deficient plants, while in Fe-supplied plants, bicarbonate stimulated FCR activity. These results are consistent with a previous study in cucumber and sunflower (Romera *et al.*, 1992*a*). Together, these results explain conflicting reports in the literature, where bicarbonate treatment decreased FCR activity (Romera *et al.*, 1997; Lucena *et al.*, 2007; García *et al.*, 2014) or stimulated FCR activity (Waters and Troupe, 2012), depending on Fe supply.

### Root molecular response to alkalinity and Fe supply

Alkalinity up-regulated many of the Fe deficiency responses even when plants were grown with normal Fe supply. While the cucumber transcription factor genes did not respond to alkaline pH at 10 μM Fe, the Fe uptake genes were up-regulated (Fig. 3). In Fe-deficient Arabidopsis, AtbHLH38 and AtbHLH39 proteins interact with AtFIT, and the resulting heterodimer regulates transcription of downstream genes like *AtFRO2* and *AtIRT1* (Yuan *et al.*, 2008). The difference in expression between transcription factors and their targets suggests that there could be regulators in addition to FIT/bHLH that respond to alkaline pH to up-regulate the expression Fe uptake genes in Fe replete, alkaline stressed plants. Alternatively, FIT or subgroup Ib bHLH protein activity (Meiser *et al.*, 2011; Hindt and Guerinot, 2012), rather than transcriptional regulation, may respond to alkaline pH at normal Fe supply.

While melon and cucumber FCR activity responded to Fe supply only at normal pH (Figs 1 and 6), cucumber Fe uptake gene expression responded to Fe deficiency at both normal and alkaline pH (Fig. 3). At low Fe supply, most of the cucumber Fe uptake genes (except *CsFIT* and *CsNRAMP1*) responded to alkaline treatment by a further increase in transcript abundance (Fig. 3). This was not the case in melon, which had diminished Fe uptake gene expression in response to Fe deficiency at alkaline pH (Fig. 6), similar to previous results in Arabidopsis (García *et al.*, 2014). In a field setting in which plants suffer from IDC, both low Fe supply and alkaline pH occur. In a prior study, low levels of bicarbonate increased expression of *CsFRO1* and *CsIRT1* in Fe-deficient roots (as seen here), while higher levels decreased expression of these Fe uptake genes (Lucena *et al.*, 2007; García *et al.*, 2014). The bicarbonate concentration we chose is in the stimulatory range for cucumber, but is in the inhibitory range for melon. In Fe-deficient plants higher concentrations of bicarbonate were required to inhibit Fe uptake gene expression in Arabidopsis than in cucumber (Lucena *et al.*, 2007). Extending this difference in response between Arabidopsis and cucumber, and cucumber and melon to other dicot species, these results provide insight into why some species of plants are more sensitive to IDC than others.

### Fe deficiency and alkaline stress regulation of riboflavin synthesis genes

Cucumber and melon roots produce and release flavin compounds in response to Fe deficiency (Welkie, 2000; Rodríguez-Celma *et al.*, 2011*a*). In cucumber and WT melon roots, most of the riboflavin synthesis genes were up-regulated by Fe deficiency in both normal pH and alkaline media (Figs 5 and 8, and Table 1). Bicarbonate treatment slightly increased expression of two or three (depending on species) of the riboflavin synthesis genes, but only at full Fe supply, and there was no synergistic up-regulation at the combined low Fe and alkaline treatment as was seen for Fe uptake genes. These results indicate that although alkaline stress induces some of the same gene expression responses as Fe deficiency, alkaline stress is not precisely equivalent to Fe deficiency stress. The overlapping but not equivalent effect of Fe deficiency and alkaline stress was seen in results of a recent metabolomics study that showed that abundance of some common metabolites changed in roots treated with low Fe or with alkaline solution, while abundance of other metabolites changed only in one of these conditions (Schmidt *et al.*, 2014). In *Medicago truncatula*, several genes and proteins of the riboflavin synthesis pathway were up-regulated in Fe-deficient roots (Rodríguez-Celma *et al.*, 2011*a*, *b*, 2013*b*). Iron-deficient *Medicago* combined with alkaline stress had increased abundance for one riboflavin synthesis protein and decreased abundance for another riboflavin synthesis protein, relative to Fe-deficient plants at normal pH (Rodríguez-Celma *et al.*, 2011*a*), and accumulated more flavins than Fe-deficient plants at normal pH, although release of flavins from roots was decreased at alkaline pH (Rodríguez-Celma *et al.*, 2011*b*). When the Arabidopsis *bHLH38* and *bHLH39* genes were overexpressed in tobacco, the plants produced more riboflavin (Vorwieger *et al.*, 2007). Our results provide a mechanism for increased riboflavin synthesis in melon and cucumber under Fe deficiency, and suggest that plants that are under alkaline stress but with normal Fe supply may also produce more riboflavin.

### Alkaline stress stimulation of Fe uptake gene expression depends on the *FeFe* regulatory pathway

Most of the Fe uptake-related genes were not regulated by Fe deficiency in the *fefe* mutant, as expected, and most of these genes also were not responsive to alkaline stress (Fig. 7B, D, H, L), suggesting that the alkaline stress signal depends mainly on the *FeFe* regulatory pathway. However, *CmHLH101* and *CmIRT1* were up-regulated in the *fefe* mutant by alkaline stress under full Fe supply (Fig. 7F, J), suggesting that alkaline stress can at least partially regulate these genes independently of the *FeFe* regulatory pathway. These genes were not induced in *fefe* mutants by alkaline stress under low Fe supply, which suggests that, as for other genes, the combination of Fe deficiency and alkaline stress may result in inhibition of Fe uptake gene expression.

Many of the Fe uptake genes that were up-regulated by alkaline stress in melon and cucumber have homologs in Arabidopsis that are targets of the primary Fe homeostasis transcription factor FIT (Colangelo and Guerinot, 2004). The expression of the *F6’H1* gene that is required for synthesis of phenolic root exudates also depends on FIT (Schmid *et al.*, 2014). The melon *FeFe* gene has not been identified, but is predicted to be a transcription factor that is functionally upstream of CmFIT (Waters *et al.*, 2014). Since melon is a flavin producer rather than a phenolics producer like Arabidopsis, we were able to test whether this primary Fe homeostasis regulator is required for expression of riboflavin synthesis genes, and whether alkaline stress responses require the *FeFe* gene. The three riboflavin synthesis genes that were up-regulated by Fe deficiency in wild-type melon (*CmRIBA1*, *CmPHS1*, and *CmDMRLs*) were dependent on FeFe for their up-regulation by Fe deficiency (Fig. 8). Combined with results for Fe uptake genes, these results show that FeFe is a master regulator of both Fe uptake genes and Fe deficiency-induced riboflavin synthesis. These results are consistent with an early characterization of the *fefe* mutant, where riboflavin efflux into the nutrient solution was increased by Fe deficiency in the wild-type, but was not increased in the *fefe* mutant (Welkie, 1996), and with our previous RNAseq results (Waters *et al.*, 2014). Thus, both flavin (melon) and phenolic (Arabidopsis) producing Strategy I species depend on master Fe uptake regulators for increased synthesis and efflux of root exudates. Up-regulation of riboflavin synthesis genes in melon roots by alkaline stress in Fe-sufficient plants also was abolished in the *fefe* mutant (Table 1 and Fig. 8), demonstrating that, like the Fe deficiency signal, alkaline stress regulation of riboflavin synthesis genes depends on the *FeFe* regulatory pathway. There is evidence for a role for both phenolics and flavins in Fe uptake under alkaline stress (Rodríguez-Celma *et al.*, 2013*b*; Schmidt *et al.*, 2014; Schmid *et al.*, 2014), which may explain why the phenolic and riboflavin synthesis genes are regulated by Fe uptake transcription factors.

### Whole plant responses to Fe supply and alkaline stress: implications for intraplant signaling and IDC

Alkaline stress may have increased expression of Fe deficiency up-regulated genes because alkaline treatment resulted in lower plant Fe accumulation. Despite increased expression of *CsFRO1* and *CsIRT1* (Fig. 3D, E) and FCR activity in roots of plants treated with full Fe supply and bicarbonate, whole plant Fe content was lower (Fig. 2A) and was similar to that of Fe-deficient plants, indicating that Fe uptake was inhibited by alkaline stress, consistent with previous studies (Fleming *et al.*, 1984; Alhendawi *et al.*, 1997). Leaf chlorophyll was also lower at all Fe levels in alkaline stressed plants (Figs. 1B and 2A) and so was Fe in stem+cotyledon and roots, while root Zn and Mn increased. These results suggest that normal Strategy I root Fe uptake processes (e.g. FCR activity, Fe(II) transporter function) do not function properly when plants are growing at alkaline pH. FCR activity of sugar beet was inhibited by alkaline pH of the assay medium (Susin *et al.*, 1996). While our FCR assay at pH 5.5 indicated that FCR activity was increased, the alkaline pH of the growth medium may disrupt the ability of the FCR protein to reduce Fe, and may inhibit other aspects of root Fe uptake, such as specificity of Fe over Zn and Mn (George *et al.*, 2012). These results extend the previous evidence that alkaline stress blocks Fe uptake and translocation.

The results discussed so far show that cucumber roots up-regulated certain Fe uptake and riboflavin synthesis genes in response to Fe limitation and alkaline stress in roots. In contrast, in cucumber leaf none of the Fe uptake genes or riboflavin synthesis genes were up-regulated in response to alkaline stress. The expression of *CsIRT1*, *CsbHLH101*, and *CsbHLH38* was up-regulated by Fe deficiency in leaf tissue, but this up-regulation was completely inhibited by bicarbonate (Fig. 4A–C), indicating that alkaline stress blocks Fe deficiency responses in leaves. It has long been suggested that alkaline stress inhibits Fe uptake into leaf cells (Mengel, 1994). Our results suggest that this lack of uptake could be caused by a lack of Fe uptake gene expression rather than simply by physical effects, such as alkalization of xylem sap pH. This abolition of gene up-regulation by Fe deficiency under alkaline stress was also true for homologs of Arabidopsis genes that are not known to be involved in Fe uptake but were up-regulated by Fe deficiency in leaves (*5960* and *KCS*; Fig. 4D, E). Thus, this inhibition of Fe deficiency up-regulated genes by alkaline stress occurs more generally than only for Fe uptake genes. These results show that the leaf responded to alkaline stress quite differently from the root, and this may have important implications for whole-plant Fe sensing.

**Fig. 9.**
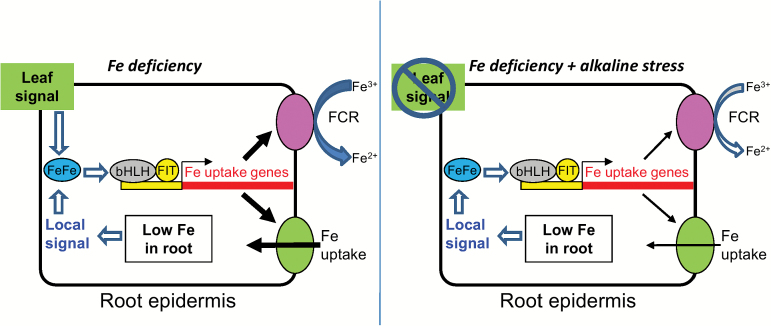
Model of responses to Fe deficiency and Fe deficiency plus alkaline stress. A local root signal for Fe deficiency acts through the FeFe regulatory pathway upstream of FIT and a bHLH partner, which activate Fe uptake genes and subsequent Fe uptake activity. An Fe deficiency signal from the leaf up-regulates the same pathway in an additive manner (left). When alkaline stress is combined with Fe deficiency (right), it blocks the normal Fe deficiency response in leaf, resulting in a loss of shoot-to-root signal. Alkaline stress combined with Fe deficiency also weakens root Fe uptake responses at the transcript and protein activity levels.

Intraplant signaling of Fe status is not well understood, but both local sensing of root Fe status and shoot-to-root signaling of leaf Fe status are important for up-regulation of root Fe uptake responses (Romera *et al.*, 1992*b*; Vert *et al.*, 2003; Lucena *et al.*, 2006; Gayomba *et al.*, 2015) through the FIT regulatory pathway. Our results give new clues into why alkaline stress may stimulate root Fe uptake responses in Fe-sufficient plants, but inhibit these responses in Fe-deficient plants. In bicarbonate-treated cucumber plants with full Fe supply, lower root Fe concentration may stimulate a local signal to increase root FCR activity and Fe uptake gene expression. The lack of response to Fe deficiency in bicarbonate-treated cucumber leaf (Fig. 4) suggests that alkaline stress interferes with leaf Fe sensing, which would be expected to inhibit shoot-to-root signaling. Since leaf signaling may be a major factor in up-regulation of root Fe uptake responses (Enomoto and Goto, 2008; García *et al.*, 2013), a lack of signal from alkaline-stressed, Fe-deficient leaves may explain why plants grown at alkaline pH did not increase root FCR activity at low Fe supply beyond the activity at full Fe supply. That is, the local Fe status root signal was already fully activated by decreased root Fe concentration resulting from alkaline stress, but the leaf signal to further increase root FCR activity was absent. This model (Fig. 9) would also explain why both alkaline and Fe deficiency signals rely mainly on the *FeFe* regulatory pathway upstream of FIT (Figs. 7 and 8).

What is not clear from our current knowledge is why leaves exposed to alkaline stress fail to respond appropriately to low Fe supply, or why Fe-deficient roots cannot maintain up-regulated FCR activity and Fe uptake gene expression (Figs. 3, 4 and 7, and Lucena *et al.*, 2007) under alkaline stress. Because a slight induction of FCR activity (Fig. 6) and *CmbHLH101* and *CmIRT1* up-regulation occurred in *fefe* mutant plants treated with full Fe supply and bicarbonate, there may be a direct pH signal that interacts with Fe status signaling. Further research into mechanisms of leaf Fe sensing and shoot-to-root signaling, and plant sensing of rhizosphere pH will be necessary to fully understand the IDC phenomenon that occurs in low Fe availability, alkaline soils. However, since plants respond to Fe deficiency differently when also exposed to alkaline stress, this study indicates that knowledge of plant responses to low Fe supply alone, or alkaline stress alone, will not be adequate to understand IDC. Future molecular physiological IDC studies will need to include Fe supply and pH treatments singly and in combination to fully understand the IDC phenomenon, and will need to incorporate leaf and root measurements simultaneously.

## Supplementary data

Supplementary data are available at *JXB* online.

Figure S1. DW of cucumber plants.

Figure S2. Cucumber (cv Miniature White) leaf chlorophyll and ferric chelate reductase activity in response to Fe supply in normal pH or alkaline nutrient solution.

Table S1. Primers used in this study.

Supplementary Data
