# Supplementary material for: Alkaline stress and iron deficiency regulate iron uptake and riboflavin synthesis gene expression differently in root and leaf tissue: implications for iron deficiency chlorosis
Source: J Exp Bot. 2016 Sep 7;67(19):5671–85. doi: 10.1093/jxb/erw328 (PMC5066488; doi:10.1093/jxb/erw328)
Supplement: Supplementary Data [file supp_67_19_5671__index.html]

Alkaline stress and iron deficiency regulate iron uptake and riboflavin synthesis gene expression differently in root and leaf tissue: implications for iron deficiency chlorosis — Alkaline stress and iron deficiency regulate iron uptake and riboflavin synthesis gene expression differently in root and leaf tissue: implications for iron deficiency chlorosis — Supplementary Data 

# Alkaline stress and iron deficiency regulate iron uptake and riboflavin synthesis gene expression differently in root and leaf tissue: implications for iron deficiency chlorosis

## Supplementary Data

Data files

- Supplementary\_Table\_S1\_Supplementary\_Figures\_S1\_S2.pdf - Supplementary Data
