# Supplementary material for: Alkaline stress and iron deficiency regulate iron uptake and riboflavin synthesis gene expression differently in root and leaf tissue: implications for iron deficiency chlorosis
Source: J Exp Bot. 2016 Sep 7;67(19):5671–85. doi: 10.1093/jxb/erw328 (PMC5066488; doi:10.1093/jxb/erw328)
Supplement: Supplementary Data [file supp_erw328_Supplementary_Table_S1_Supplementary_Figures_S1_S2.pdf]

Alkaline stress and iron deficiency regulate iron uptake and riboflavin synthesis gene expression differently in root and leaf tissue: Implications for iron deficiency chlorosis (IDC)

En-Jung Hsieh and Brian M. Waters

Supplementary data

Table S1. Primers used in this study.

Gene name	Primer sequence 5' → 3'
Ubiquitin F	TCTTTCTTGGCCTCGTGCTCTTCT
Ubiquitin R	TTGCAGATGCGTGGAATCGACAAC
CsbHLH38 F	GTGGAAGAGCAGATGAGGAAG
CsbHLH38 R	TGCGGAAATGATCGATGAGG
CsbHLH101 F	GTGCTGTGAATTGGCTTAGTG
CsbHLH101 R	AGGATCGAAAAGAGGAAGCAG
CmFRO1 F	TCACAGCGATTTAGAACCAGA
CmFRO1 R	GCCTTCGAGGGAAACTTGAA
CmFIT F	GACATCAACGATCAATTTGAGC
CmFIT R	CGATCCTCGATCAAGCAAC
CmIRT1 F	AAACCGGTGGCGAGAATGATACCT
CmIRT1 R	ACTCCAATGTTGCACCCGGATAGA
CmNRAMP1 F	CTGTAATAGCAGCCGATATTCC
CmNRAMP1 R	CTATCAACAGTTCAGCTTCC
CsRIBA1 F	TGAAGCCTCTGTCGACCTTG
CsRIBA1 R	CGAAGCTTGGGGAGTCTAGC
CsCmPYRD F	GGCGTGCAACGACTAAGAGA
CsCmPYRD R	GTGAGAAGAGGCTTCCCAGTC
CsPHS1 F	GCCTCCTTGTTAATGCTCCA
CsPHS1 R	CGATGTCGAGATGTAACGCT
CsDMRLs F	GGTCCCAGGGAGCTTTGATA
CsDMRLs R	ACAACGGCATCATAGTGGGA
Cm bHLH38 F	AACAACAAGTGGAAGGGCAG
Cm bHLH38 R	CGAAGCTGCGGATTTCATTTT
Cm bHLH101 F	TCCAGAGCTTCAACAGCAAG
Cm bHLH101 F	AGCACACAAAGAACTCATCCA
CmRIBA1 F	TGGCATTGGCATCAAAACAC
CmRIBA1 R	GCCTTTTCAAAACCCCTCCT
CmPHS1 F	CGAGGAAGAAGCGATGCTAT
CmPHS1 R	ACATCACAACACGAACAGGA
CmDMRLs F	AGAAAGTCAAGCGAGGAGAAT
CmDMRLs F	GAGGCCGCCATTATCAACTC
CsCmRIBC	ACTGCTTTTGACCACCAACT
CsCmRIBC	CCATTCGGCTGATTGGTTGA

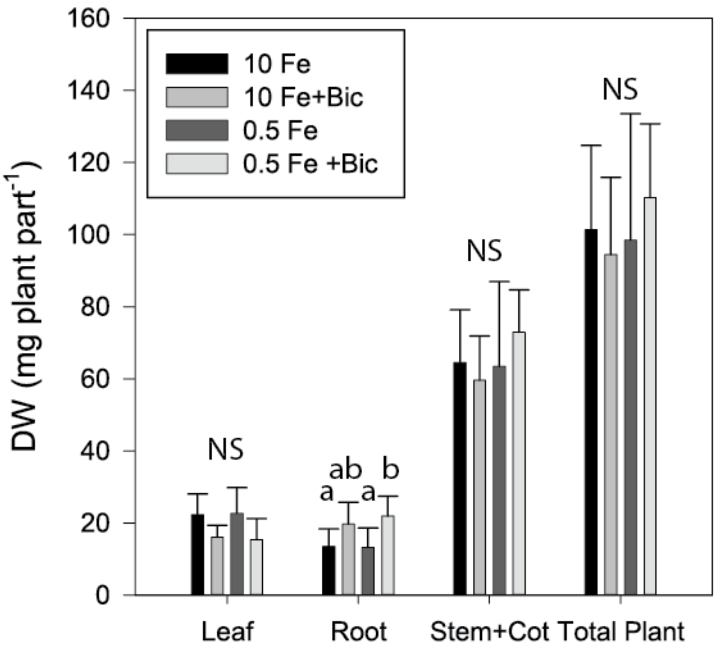

Fig. S1. DW of cucumber plants. Cucumber (cv. Ashley) seedlings were transferred into hydroponics with 0.5 or 10 μM Fe for 4 days pretreatment, and then treated with or without 10 mM bicarbonate for 3 days. The roots, first leaf, and stem+cotyledons of each plant were harvested separately and DW was measured. Total Plant DW is the sum of plant parts. Bars represent mean ± s.d. (n=8). Different letters indicate significant differences ($P < 0.05$) based on ANOVA using the Holm-Sidak method, NS indicates no significant differences between treatments.

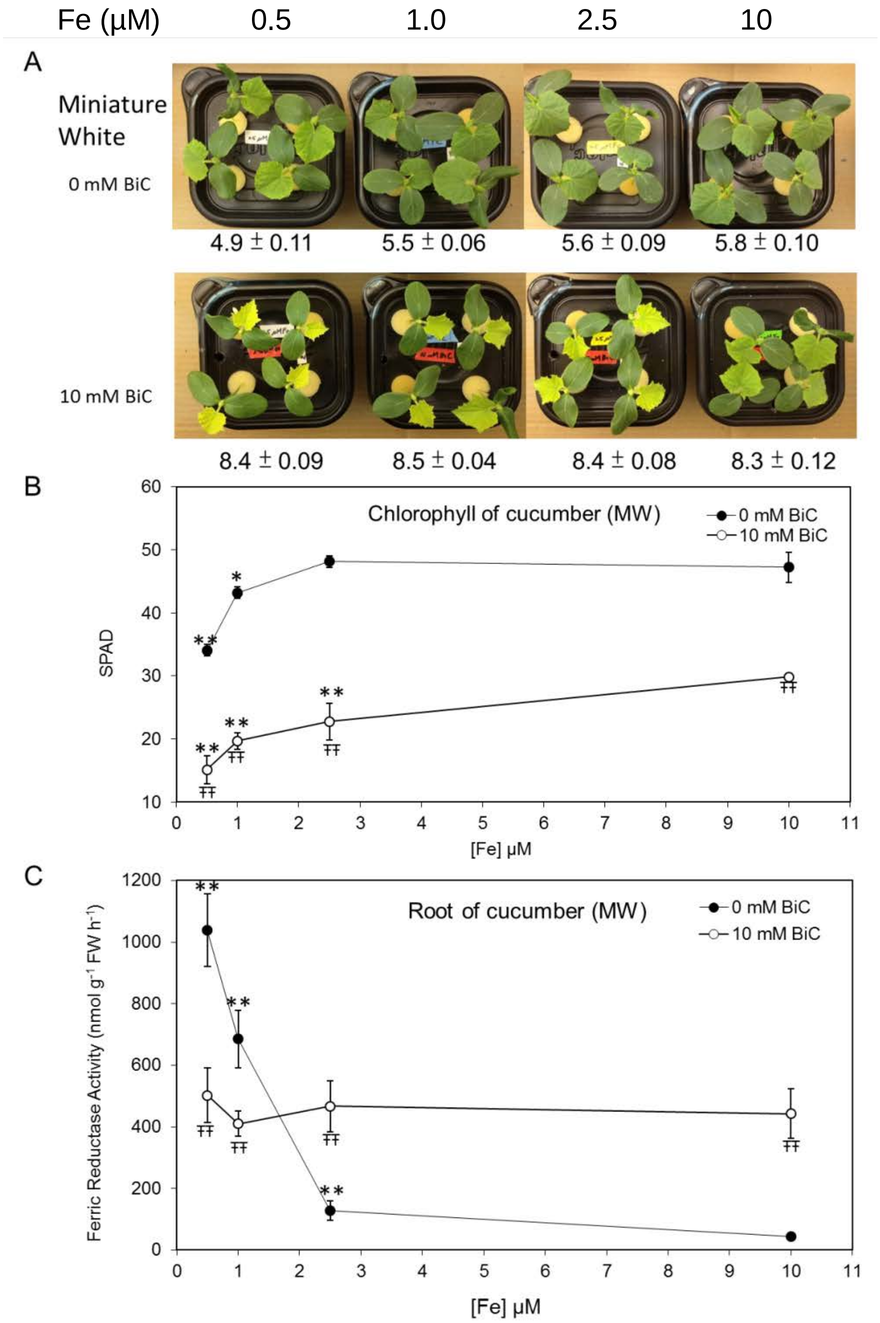

Fig. S2. Cucumber (cv Miniature White) leaf chlorophyll and ferric-chelate reductase activity in response to Fe supply in normal pH or alkaline nutrient solution. Cucumber seedlings were pre-treated in hydroponic solution for 4 d with 0.5, 1.0, 2.5, or 10 µM Fe, and then supplied without or with 10 mM bicarbonate for 3 d. (A) Photograph of the plants in each treatment, with final solution pH (means ± SD, n=6) indicated below photograph. (B) Chlorophyll level of first leaf as measured by Minolta SPAD chlorophyll meter of 10-day-old cucumber plants. (C), Ferric chelate reductase activities of roots, measured after 3 days of treatment. *, ** indicate statistical significance of $P < 0.05$, $P < 0.01$, respectively, compared to 10 µM Fe within each curve,. Ŧ, ŦŦ indicate statistical significance of $P < 0.05$, $P < 0.01$, respectively, comparing 10 mM bicarbonate to 0 mM bicarbonate at each Fe supply.
